# Psychological Stress Delays Periodontitis Healing in Rats: The Involvement of Basic Fibroblast Growth Factor

**DOI:** 10.1155/2012/732902

**Published:** 2012-12-30

**Authors:** Ya-Juan Zhao, Qiang Li, Bai-Xiang Cheng, Min Zhang, Yong-Jin Chen

**Affiliations:** Department of General Dentistry and Emergency, School of Stomatology, Fourth Military Medical University, No. 145 Changle West Road, Xi'an, Shaanxi 710032, China

## Abstract

*Objective*. To evaluate the effects of psychological stress on periodontitis healing in rats and the contribution of basic fibroblast growth factor (bFGF) expression to the healing process. *Methods*. Ninety-six rats were randomly distributed into control group, periodontitis group, and periodontitis plus stress group. Then, the rats were sacrificed at baseline and week(s) 1, 2, and 4. The periodontitis healing condition was assessed, and the expression of interleukin-1**β** (IL-1**β**), tumor necrosis factor-**α** (TNF-**α**), and bFGF were tested by immunohistochemistry. *Results*. The stressed rats showed reduced body weight gain, behavioral changes, and increased serum corticosterone and ACTH levels (*P* < 0.05). The surface of inflammatory infiltrate, alveolar bone loss, attachment loss, and expression of IL-1**β** and TNF-**α** in the stress group were higher than those in the periodontitis group at weeks 2 and 4 (*P* < 0.05). Rats with experimental periodontitis showed decreased bFGF expression (*P* < 0.05), and the recovery of bFGF expression in the stress group was slower than that in the periodontitis group (*P* < 0.05). Negative correlations between inflammatory cytokines and bFGF were detected. *Conclusion*. Psychological stress could delay periodontitis healing in rats, which may be partly mediated by downregulation of the expression of bFGF in the periodontal ligament.

## 1. Introduction

Periodontitis is defined as the inflammation of periodontal supporting tissue caused by specific pathogenic microorganisms, resulting in the advanced destruction of the periodontal ligament and the alveolar bone and subsequently leading to the formation of a gingival recession and a periodontal pocket [1]. Although the bacteria and plaque are the initiating factors of periodontitis, a simple bacterial infection is not sufficient to explain the range of severity of this condition in different individuals. In recent years, it has become accepted that susceptibility to periodontitis varies greatly between individuals who harbor the same pathogenic microflora [2]. Epidemiological studies have found a positive correlation between psychological stress and periodontal disease, suggesting that stress, depression, or anxiety, as systemic risk factors, may modify the host response and further contribute to the occurrence of periodontal disease [3–5]. 

Periodontitis is a chronic inflammatory disease characterized by episodes of active destruction in which pronounced inflammatory infiltration, attachment loss, and alveolar bone loss occur as well as periods of quiescence in which periodontal regeneration takes place, including the restoration of the damaged periodontal ligament, the gingival tissue, the alveolar bone, and the cementum to their original levels [6, 7]. Inflammation of periodontal tissue is the main pathological feature in periodontitis and the tissue injury in periodontitis may be caused by the inability of the host to resolve the inflammation [6]. Studies also have shown that when periodontitis exists, some inflammatory cytokines, such as IL-1*β* and TNF-*α*, are significantly increased in periodontal tissue, gingival crevicular fluid, or peripheral blood [8, 9].

The process of periodontal healing is regulated by multiple growth factors, among which bFGF is recognized as a key factor in periodontal ligament regeneration [10–13]. As a typical member of the heparin-binding growth factor family that was first isolated from bovine pituitary extracts and shown to stimulate fibroblast proliferation, bFGF is a multifunctional factor whose activity is crucial for enhanced normal wound healing [14]. bFGF has been reported to exert a variety of effects on cell proliferation, differentiation, and angiogenesis [15]. In animal studies, the topical application of bFGF can promote periodontal healing and regeneration without epithelial downgrowth, ankylosis, or root resorption in a surgically created furcation [16, 17]. However, few studies have focused on the expression of endogenous bFGF in the periodontal ligament during the destruction and healing process of periodontitis.

More recently, several animal studies have suggested the influence of psychological stress on the susceptibility to and progression of periodontal disease, indicating that the activation of the hypothalamic-pituitary-adrenal (HPA) axis seems to play an important role [18–20]. Additionally, it is known that psychological stress can delay wound healing by affecting the host immune response [21, 22]. However, there is limited information about the effect of psychological stress on wound healing within the periodontal tissue. Thus, we hypothesized that psychological stress could delay periodontal healing through the downregulation of bFGF expression. We tested this hypothesis by establishing an animal model of experimental periodontitis with psychological stress during periodontal healing. We performed histomorphometric analysis and immunohistochemical staining of bFGF expression.

## 2. Materials and Methods

### 2.1. Animals

Ninety-six adult male Sprague-Dawley rats (provided by the Laboratory Animal Center of the Fourth Military Medical University, Xi'an, China) at 8 weeks of age and weighing 247.24 ± 11.06 g were caged in a room with controlled temperature (22 ± 1°C), humidity (60 ± 5%), and light-dark cycle (light on 8:00 to 20:00 h). The animals were given access to food and water* ad libitum*. The rats were randomly distributed into three groups, with 32 rats in each group: control rats; rats with periodontitis that underwent a natural healing process (periodontitis group); rats with periodontitis that were subjected to psychological stress during the healing process (stress group). There were four subgroups in each group that were evaluated at different observation time points: baseline (immediately after ligature removal), 1 w (1 week after ligature removal), 2 w (2 weeks after ligature removal), and 4 w (4 weeks after ligature removal). This study was performed in strict accordance with the recommendations in the Guide for the Care and Use of Laboratory Animals of the National Institutes of Health. The protocol was approved by the Animal Research Ethics Committee of the Fourth Military Medical University (Xi'an, China) and every effort was taken to avoid animal suffering at each stage of the experiment.

### 2.2. Experimental Periodontitis

The rats were anesthetized by intraperitoneal injection of 1% sodium pentobarbital (3.5 mg/Kg) prepared in sterile saline, and a 4–0 silk ligature was placed around the cervix of the right second maxillary molar to induce experimental periodontitis as previously described [19], except for the animals in the control group, which were submitted to the same procedure but without ligature placement. The ligatures were kept in place for four weeks and served as a retention device for oral microorganisms. Then, the ligatures were removed for 1, 2, or 4 weeks of followup during the spontaneous natural healing process in the periodontitis group or healing under chronic unpredictable mild stress (CUMS) in the stress group.

### 2.3. Psychological Stress

After ligature removal, the rats in the psychological stress group were subjected to CUMS which contains seven different mild psychological stressors including damp sawdust for 24 hours, food deprivation for 12 hours, water deprivation for 12 hours, inversion of the light-dark cycle, swimming in 4°C cold water for 5 min, swimming in 45°C hot water for 5 min, and 1 h of restraint stress (the rats were kept in a restrainer made of an inflexible wire mesh with a sliding door to facilitate the restraint; during the stress procedure, the rats were not allowed to move freely, but their bodies were not constricted) [23, 24]. Over the course of each week of the CUMS procedure, one of the 7 stressors was applied each day on a random schedule. The same stressor appeared only once per week to avoid stress habituation in rats. At baseline and at weeks 1, 2, or 4, the rats (*n* = 8 rats each) were weighed and prepared for the subsequent tests. The body weight gain was calculated according to the formula: [(Body weight at time point *t*) − (Initial body weight)]/(Initial body weight), where the time point is baseline or week 1, 2, or 4.

### 2.4. Open-Field Test

The open-field chamber (RD 1412-OF, Shanghai Mobile datum Corporation, Shanghai, China) was placed inside a temperature-controlled chamber. This chamber consists of a 100 cm × 100 cm × 80 cm Plexiglas box illuminated by one fluorescent light suspended over the chamber. The sides of the chamber were white, the bottom was brown, and the top was open to the air. The activities of each rat were automatically monitored using a digital video camera for 15 min in the open field. After each test, the maze was thoroughly cleaned with 20% alcohol to eliminate the odor and trace of the previously tested animal. The distance moved in center and the total distance moved were calculated to assess the animal's stress condition [25].

### 2.5. Sample Collection

The rats were anesthetized by intraperitoneal injections of 1% sodium pentobarbital (3.5 mg/Kg) prepared in sterile saline. Next, blood samples were collected (1.5–2 mL from each animal) by cardiac puncture. The blood was allowed to coagulate on ice for 1 h. Thereafter, the serum was isolated following refrigerated centrifugation (at 4°C) for 30 min at 3000 rpm. After blood sample collection, the rats were sacrificed by cervical dislocation. The right maxillae were immediately removed and fixed in 10% formalin for >48 hours. Subsequently, decalcification was carried out in 15% EDTA solution at room temperature for 6 weeks. Serial paraffin sections that were 5 *μ*m thick were obtained in the mesial-distal direction and stained with hematoxylin and eosin (H&E) for histologic analysis.

### 2.6. Measurement of Serum Corticosterone and ACTH

The serum aliquots were aspirated and stored in microcentrifuge tubes at −20°C for measurement. The serum corticosterone (CORT) and adrenocorticotropic hormone (ACTH) levels were determined with commercial enzyme-linked immunosorbent assay (ELISA) kits (Shanghai Xitang Bioengineering Institute, Shanghai, China) as previously described [26].

### 2.7. Histomorphometric Analysis

**Figure 1:**
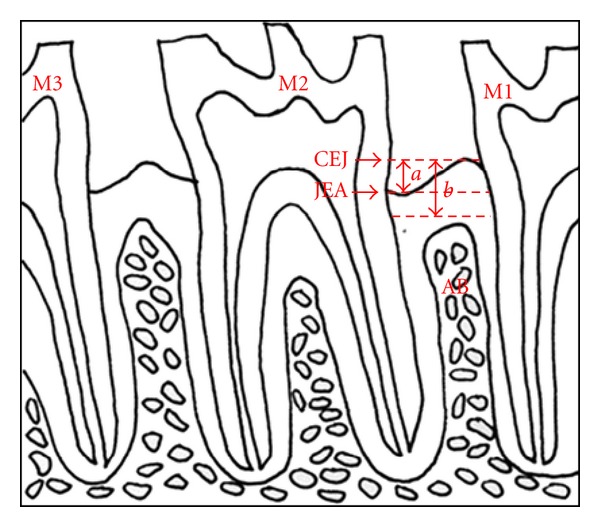
Schematic illustration of the histometric parameters evaluated. M1: maxillary first molar; M2: maxillary second molar; M3: maxillary third molar; CEJ: cement-enamel junction; JEA: junctional epithelial attachment; AB: alveolar bone; *a*: attachment loss; *b*: alveolar bone loss.

The histomorphometric analysis was performed under a light microscope at a magnification of ×100. The measurements included evaluation of the inflammatory infiltrate by measuring the surface of the connective tissue infiltrated with inflammatory cells (SI), evaluation of the alveolar bone loss (ABL) by measuring the distance between the cement-enamel junction and the most coronal border of the alveolar bone, and evaluation of the attachment loss (AL) by measuring the distance between the cement-enamel junction and the epithelial attachment [19] Figure 1.

### 2.8. Immunohistochemical Staining

Immunohistochemical staining was performed using a three-step avidin-biotin complex method as previously described [27, 28]. The primary antibodies were rabbit polyclonal antibody to IL-1*β* (diluted at 1 : 200, Abcam Biotechnology, Cambridge, USA), TNF-*α* (diluted at 1 : 200, Abcam Biotechnology, Cambridge, USA), and bFGF (diluted at 1 : 50, Abcam Biotechnology, Cambridge, USA). The observation area was photographed using a light microscope with a magnification of ×200 and ×400. (Nikon Microphoto-FXA, Tokyo, Japan). Areas comprising 100 × 200 *μ*m sections of the periodontal ligament of the mesial root surface of the maxillary second molar, which were located just below the crest of the alveolar bone, were used for investigation. According to the histological observations and preliminary data, the number and the area of bFGF-immunoreactive cells per unit area were recorded [27] using image analysis software (Image-Pro Plus, Media Cybernetics, USA). The signal intensity was measured [29] using a high-resolution pathological image analysis system (Image-Pro Plus, Media Cybernetics, USA), and the optical density of each section was obtained.

### 2.9. Statistical Analysis

 The experimental data were expressed as the means ± standard error of means (SEM). Differences among the groups were analyzed by one-way analysis of variance (ANOVA) with the SPSS 13.0 software (SPSS Institute, Chicago, IL, USA) followed by the SNK-q test for multiple comparisons. Correlation between variables was calculated using the Spearman test. *P* < 0.05 was considered statistically significant for all analyses.

## 3. Results

### 3.1. Body Weight Gain

**Figure 2:**
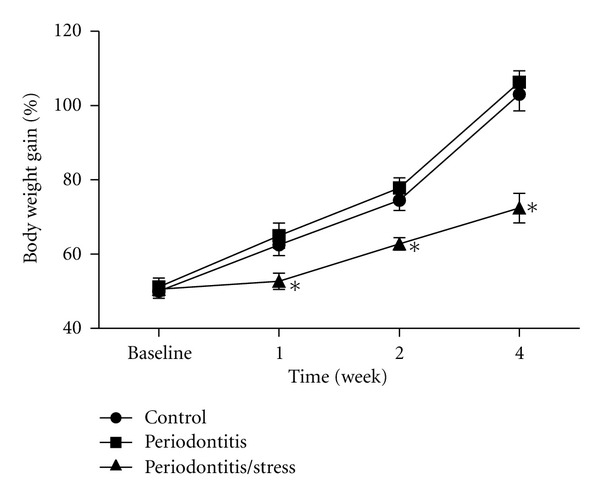
Body weight gain of the rats in all groups. *P < 0.05 versus control group at the same time point.

The body weight gain of the rats in the stress group was significantly lower than that in the control group at weeks 1, 2, and 4 (*P* < 0.05, Figure 2), whereas no significant difference was observed between the periodontitis group and the control group at any time point (*P* > 0.05, Figure 2).

### 3.2. Behavioral Changes

**Figure 3:**
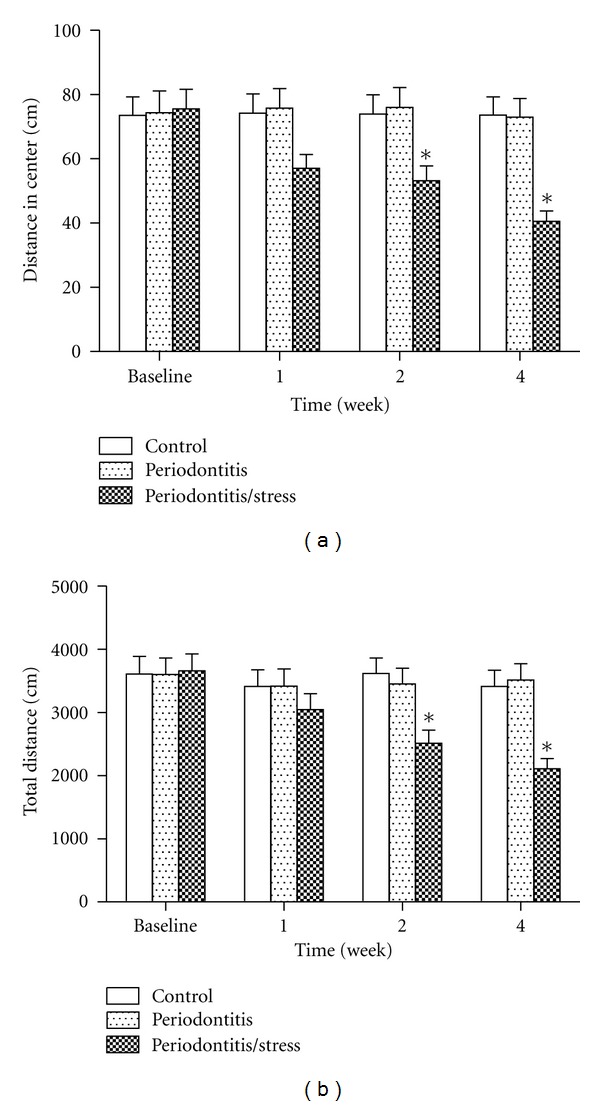
Effect of psychological stress on behavioral changes of rats in the open-field test. The distance moved in the center (a) and total distance moved (b) were measured. *P < 0.05 versus matched control group for the same time point.

In the open-field test, the distance moved in the center and total distance moved by the rats in the stress group were significantly less than the distances moved by the control group at weeks 2 and 4, whereas no significant difference was observed between the stress group and the control group at week 1 (*P* < 0.05, Figures 3(a) and 3(b)). There was also no significant difference between the periodontitis group and the control group at any point (*P* > 0.05, Figures 3(a) and 3(b)).

### 3.3. Serum CORT and ACTH Levels

**Figure 4:**
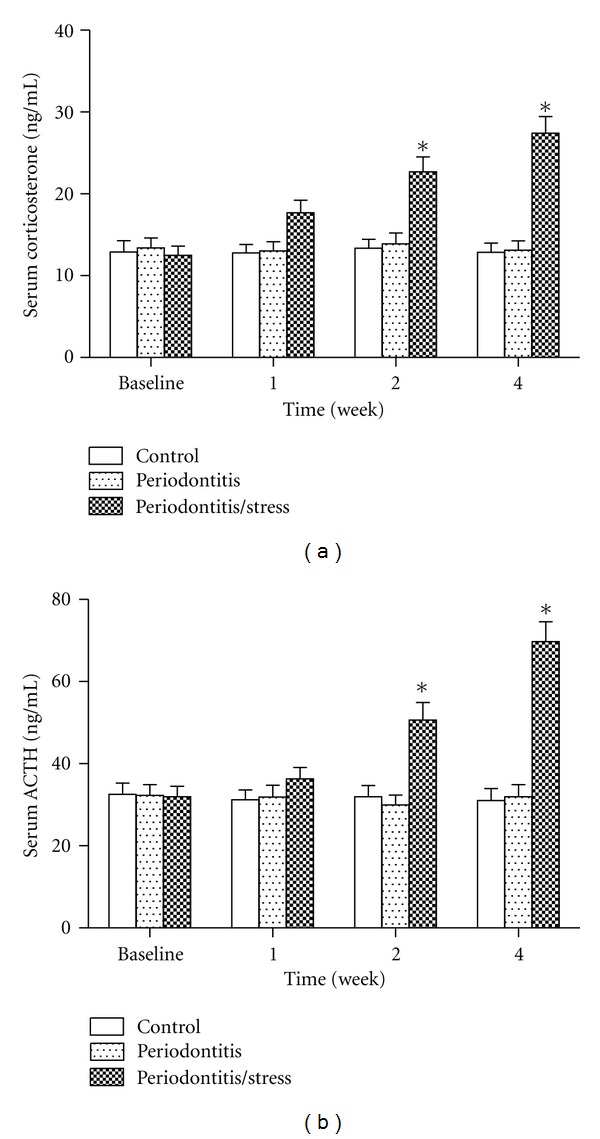
Serum corticosterone level (a) and serum ACTH level (b) of the rats in all groups. *P < 0.05 versus control group of the same time point.

The serum corticosterone levels of the rats in the stress group were significantly higher in the control group at weeks 2 and 4, but not at week 1 (*P* < 0.05, Figure 4(a)). Such a difference was also observed in the serum ACTH levels (*P* < 0.05, Figure 4(b)). In contrast, no significant difference was observed between the periodontitis group and the control group at any time point (*P* > 0.05, Figures 4(a) and 4(b)).

### 3.4. Histometric Findings

**Figure 5:**
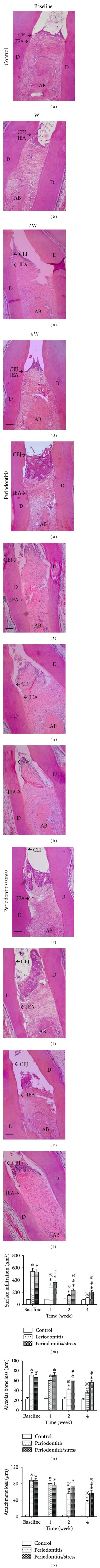
H&E staining. ((a)–(l)) Area comprising the distal root of the maxillary first molar and the mesial root of the maxillary second molar of all groups at all time points (D: dentin, AB: alveolar bone, CEJ: cement-enamel junction, and JEA: junctional epithelial attachment, ×100, bar represents 150 *μ*m). ((m)–(o)) Histologic analysis of the surface of inflammatory infiltration (m), alveolar bone loss (n), and attachment loss (o) of the rats in all groups. *P < 0.05 versus control group at the same time point. ^#^ *P* < 0.05 versus periodontitis group at the same time point. ^*※*^ *P* < 0.05 versus the same group at baseline.

 In the present study, the time point when the periodontitis group and periodontitis/stress group rats having experienced 4-week ligature placement was chosen as baseline to fully explore the effects of psychological stress on periodontitis healing. That is, at baseline, the periodontitis group contained the rats suffering periodontitis and, further to experience the natural healing process, the periodontitis/stress group contained the rats suffering periodontitis and further to experience the healing process under CUMS, and the blank control group contained the healthy rats without experimental periodontitis. The results showed that in the control group at each time point, the invasion of inflammatory cells was confined to the area beneath the epithelium. Moreover, minimal alveolar bone loss and attachment loss were observed (Figures 5(a)–5(d)). In the periodontitis group and the stress group at the baseline observation point, there was a significant periodontal breakdown, including pronounced inflammatory infiltration, mostly composed of mononuclear cells with a few polymorphonuclear leukocytes, significant alveolar bone loss, and attachment loss (Figures 5(e) and 5(i)). The data analysis indicated that inflammatory infiltrate (SI), alveolar bone loss (ABL), and attachment loss (AL) were significantly increased compared with the matched control group (*P* < 0.05, Figures 5(m)–5(o)). These results confirmed that experimental periodontitis was successfully induced by 4-week ligature placement. 

 After ligature removal, the rats in the periodontitis group showed spontaneous healing of the soft tissue and remodeling of the alveolar bone in the following four weeks, whereas psychological stress resulted in significantly delayed healing of the periodontal tissue (Figures 5(f)–5(h) and 5(j)–5(l)). Histometric analysis showed that there was a significant reduction of SI, ABL, and AL in the periodontitis group, although these values were still higher than those in the matched control group at all time points except for SI at week 4 (*P* < 0.05, Figures 5(m)–5(o)). Moreover, SI and ABL were significantly higher in the stress group than in the periodontitis group at week 2 and week 4 (*P* < 0.05, Figures 5(m) and 5(n)). Additionally, a higher AL was observed in the stress group than in the periodontitis group at week 4 (*P* < 0.05, Figure 5(o)). 

### 3.5. Immunohistochemical Staining of IL-1*β*, TNF-*α*, and bFGF

**Figure 6:**
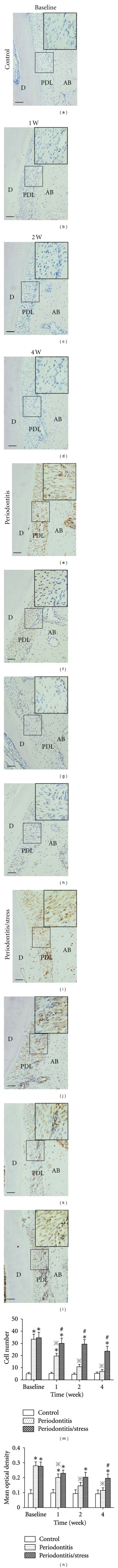
Immunostaining of interleukin-1*β*. ((a)–(l)) Area comprising the mesial root surface of the maxillary second molar of all groups at all time points (D: dentin, AB: alveolar bone, and PDL: periodontal ligament, ×200, bar represents 100 *μ*m). The boxed areas indicate a higher magnification (×400). ((m)-(n)) Quantitative analysis of the number of immunoreactive cells (m) and the intensity of staining (n). *P < 0.05 versus control group at the same time point. ^#^ *P* < 0.05 versus periodontitis group at the same time point. ^*※*^ *P* < 0.05 versus the same group at baseline.

**Figure 7:**
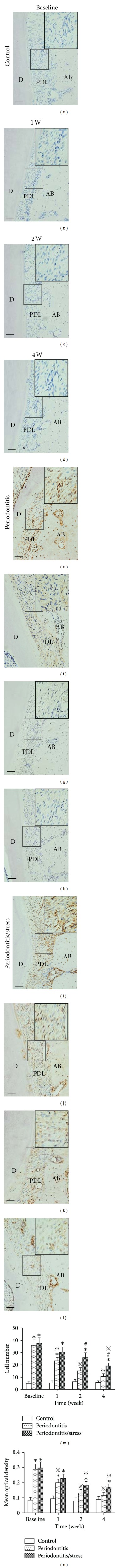
Immunostaining of tumor necrosis factor-*α*. ((a)–(l)) Area comprising the mesial root surface of the maxillary second molar of all groups at all time points (D: dentin, AB: alveolar bone, and PDL: periodontal ligament, ×200, bar represents 100 *μ*m). The boxed areas indicate a higher magnification (×400). (m)-(n)) Quantitative analysis of the number of immunoreactive cells (m) and the intensity of staining (n). *P < 0.05 versus the control group at the same time point. ^#^ *P* < 0.05 versus the periodontitis group at the same time point. ^*※*^ *P* < 0.05 versus the same group at baseline.

Regarding IL-1*β* and TNF-*α* staining, little immunoreactive cells and low-level mean optical density were found in the control group at all time points (Figures 6, 7(a)–7(d), 7(m), and 7(n)). At baseline, ligature placement resulted in a significant increase in the number of immunoreactive cells and the mean optical density in the periodontitis group and the stress group (*P* < 0.05, Figures 6, 7(e), 7(i), 7(m), and 7(n)). After ligature removal, the expression of IL-1*β* and TNF-*α* significantly decreased in the periodontitis group at week 1, 2, and 4 compared with baseline level (*P* < 0.05, Figures 6, 7(m), and 7(n)). For IL-1*β*, the immunoreactive cell number and the mean optical density did not descend significantly in the stress group compared with baseline level (*P* > 0.05, Figures 6(m) and 6(n)). Moreover, the cell number at week 1, 2, and 4 and the mean optical density at week 4 were higher in stress group than that in the periodontitis group (*P* < 0.05, Figures 6(m) and 6(n)). For TNF-*α*, although there was an expression downtrend, the immunoreactive cell number was still higher in the stress group than in the periodontitis group at week 2 and week 4 (*P* < 0.05, Figure 7(m)).

**Figure 8:**
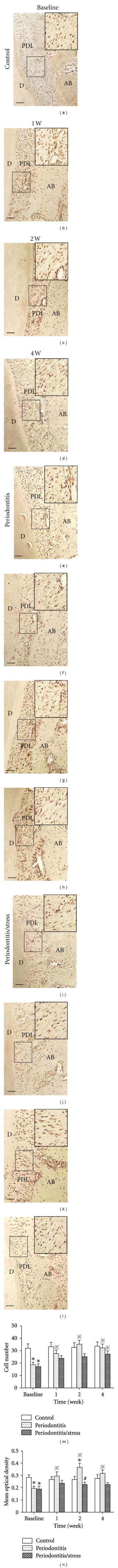
Immunostaining of basic fibroblast growth factor. ((a)–(l)) Area comprising the mesial root surface of the maxillary second molar of all groups at all time points (D: dentin, AB: alveolar bone, and PDL: periodontal ligament, ×200, bar represents 100 *μ*m). The boxed areas indicate a higher magnification (×400). ((m)-(n)) Quantitative analysis of the number of immunoreactive cells (m) and the intensity of staining (n). *P < 0.05 versus control group at the same time point. ^#^ *P* < 0.05 versus periodontitis group at the same time point. ^*※*^ *P* < 0.05 versus the same group at baseline.

As for bFGF staining, moderate immunoreactivity was found in the control group at all time points (Figures 8(a)–8(d), 8(m), and 8(n)). At baseline, ligature placement resulted in a significant reduction in the number of immunoreactive cells and the mean optical density in the periodontitis group and the stress group (*P* < 0.05, Figures 8(e), 8(i), 8(m), and 8(n)). In the following 4 weeks after ligature removal, bFGF expression in the periodontitis group prominently recovered when the cell number and mean OD were significantly increased at weeks 1, 2, and 4 (*P* < 0.05, Figures 8(f)–8(h), 8(m) and 8(n)). Furthermore, the mean OD of the periodontitis group was significantly higher than that in the control group at week 2 (*P* < 0.05, Figure 8(n)). In contrast, psychological stress significantly delayed the recovery of bFGF expression, whereas the positive cell number and the mean OD did not increase significantly (*P* > 0.05, Figures 8(j)–8(l), 8(m), and 8(n)), with the exception of the cell number at week 4 (*P* < 0.05, Figure 8(m)). 

**Figure 9:**
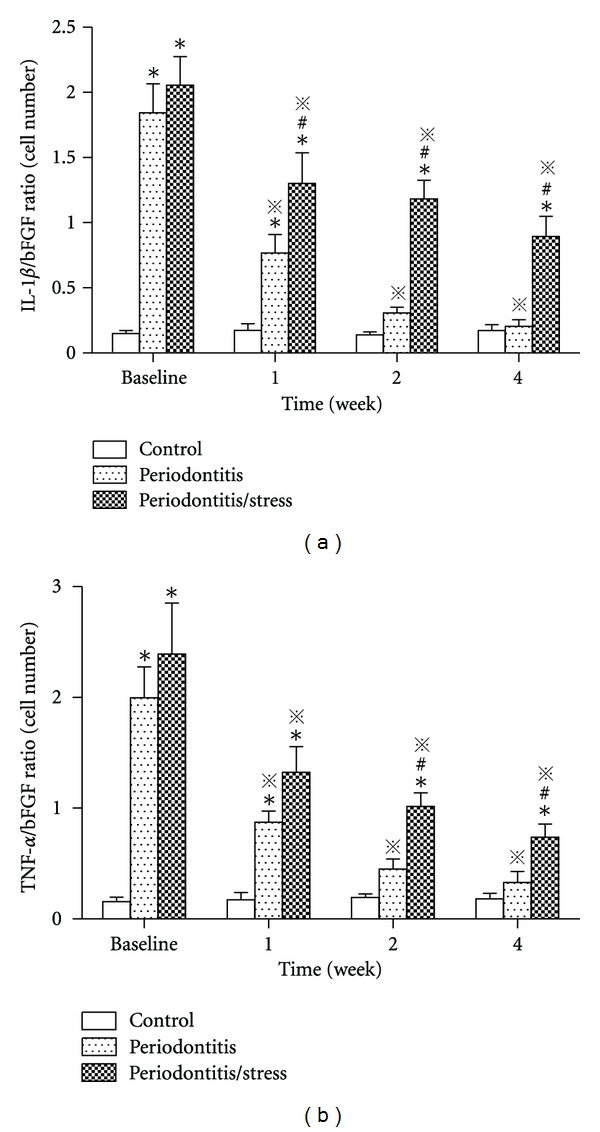
Ratios of immunoreactive cell numbers between inflammatory cytokines and bFGF during the observation period. (a) Immunoreactive cell number ratio between IL-1*β* and bFGF. (b) Immunoreactive cell number ratio between TNF-*α* and bFGF. *P < 0.05 versus control group at the same time point. ^#^ *P* < 0.05 versus periodontitis group at the same time point. ^*※*^ *P* < 0.05 versus the same group at baseline.

Further, the ratios of immunoreactive cell numbers between inflammatory cytokines and bFGF, which indicated the homeostasis between inflammation and repair process, were calculated and statistically analyzed. It showed that both the IL-1*β*/bFGF ratio and TNF-*α*/bFGF ratio were highest at baseline in periodontitis and periodontitis/stress group. As the healing time went on, the inflammatory cytokine/bFGF ratios decreased apparently (*P* < 0.05, Figures 9(a) and 9(b)). Compared with the ratios of periodontitis group, those of the periodontitis/stress group decreased significantly much slower (*P* < 0.05, Figures 9(a) and 9(b)).

In addition, the negative correlations between inflammatory cytokines and bFGF were observed (*r* = −0.539,*P* < 0.0001 for IL-1 and bFGF, and *r* = −0.542,*P* < 0.0001 for TNF-*α* and bFGF). In contrast, the positive correlations between SI and inflammatory cytokines were observed (*r* = 0.494,*P* < 0.0001 for SI and IL-1, and *r* = 0.519, *P* < 0.0001 for SI and TNF-*α*). No significant correlation was found between SI and bFGF (*r* = −0.256, *P* = 0.059)

## 4. Discussion

Previous clinical studies have demonstrated a correlation between psychological stress and a more severe level of destruction during the progression of periodontitis [4]. Animal studies have also found a higher degree of attachment loss and alveolar bone resorption after exposing the animals to experimental periodontitis and stress at the same time [18, 30]. However, from periodontitis to periodontal healing, the pathological condition of the periodontal tissue shifts from destruction to regeneration. Whether psychological stress has an adverse impact on the healing process of periodontitis and whether bFGF plays a role in that process remain unclear. Therefore, this study was designed to evaluate the effect of psychological stress on the healing process of periodontitis and the expression of bFGF in the periodontal ligament.

The experimental model of periodontitis employed in the current study is induced by the local compression of a silk ligature that results in progressive accumulation of plaque and increased degradation of periodontal connective tissues and bone resorption [31, 32]. As the previous studies have reported [19, 30, 33], we also observed a significant increase in the surface of inflammatory infiltration, alveolar bone loss, and attachment loss in periodontitis group and periodontitis/stress group compared with those of control, which confirmed a successful establishment of periodontitis in rats. In addition, the results of the present work suggested an obvious downregulation of the expression of bFGF in the periodontal ligament associated with experimental periodontitis, which was demonstrated by the decreased immunoreactive cell number and mean optical density. These results may indicate that periodontal ligament repair is diminished in the destruction phase of periodontitis. Our findings are similar to those of Gao et al. [34], who identified, for the first time, the presence of bFGF in human periodontal ligament tissue and observed a significant decrease in the fibroblast number and staining intensity of bFGF in the periodontal ligament of patients with chronic periodontitis. Moreover, the destruction of periodontal tissue may be attributed to the failure of recruitment and activation of macrophages, involving little bFGF expression [35]. In contrast, as an important angiogenesis-associated factor, bFGF is upregulated in some other oral pathologies that involve the formation of a large number of new blood vessels, such as pyogenic granuloma [36]. 

The stress model that we adopted in the current study is CUMS, which has been developed in recent years and is widely used to mimic chronic stress in daily life in studies [23, 24, 26]. The major advantage of CUMS is the avoidance of animal adaptation to invariant stressors by employing various physical and psychological stressors in a predetermined manner. We have found that the experimental animals showed a reduction in body weight gain and elevated plasma corticosterone and ACTH levels. Moreover, the rats exposed to psychological stress showed stress-like behaviors based on the significant decrease observed in the distance moved in the center and total distance moved in the open-field test. These results are in accordance with previous studies [9, 37], indicating that the experimental animals were successfully stressed by the CUMS procedure.

Some clinical studies have demonstrated a link between psychological stress and periodontitis [3–5], and several investigators have worked to determine the possible mechanisms behind it. Peruzzo et al. [30] reported the modulated expression of pro- and anti-inflammatory and proresorptive factors, suggesting that stress may alter the progression of periodontitis locally through its effect on the immune-inflammatory system. Another study conducted with animal models [33] speculated on a correlation between periodontitis severity and psychological stress and periodontal tissue hypoxia by measuring the expression of hypoxia-induced factor-1*α*. Nevertheless, there is limited information regarding whether psychological stress has an adverse effect on the healing stage of periodontitis. Thus, in our study, psychological stress was imposed on animals after ligature removal. On the one hand, in the periodontitis group, we observed spontaneous periodontal healing at 1, 2, or 4 weeks compared with baseline. This healing was characterized by a decreased surface of inflammatory infiltration, alveolar bone remodeling, and epithelial reattachment, confirming the results reported by Coimbra et al. [38], who also observed significant repair of the soft tissue and regeneration of the alveolar bone in rats 15 days after ligature removal. On the other hand, we observed delayed periodontal healing in the stress group, and the periodontal breakdown was significantly more severe in the stress group than in the periodontitis group. These findings may suggest that psychological stress could impair the healing process of periodontitis.

Periodontitis is a chronic infectious disease that the host inflammatory response to bacteria is the leading cause of tissue destruction. In our study, the expressions of two typical inflammatory cytokines, IL-1*β* and TNF-*α*, significantly increased in periodontal ligament after ligature placement, confirming the previous studies that in periodontitis the elevated levels of inflammatory cytokines can cause tissue injury and alveolar bone metabolism breakdown [29, 39]. In fact, we did find the positive correlation between inflammatory cytokines expression and periodontal damage. In the natural healing process of periodontitis, the expression of IL-1*β* and TNF-*α* reduced markedly. In contrast, during periodontitis healing under conditions of psychological stress, the expression of inflammatory cytokines remained on a relatively higher level. The results were similar to our previous study [40], which demonstrated that psychological stress could induce upregulation of IL-1*β* and TNF-*α* and may further play a potential role in initiating the cartilage destruction. The present study suggested that psychological stress may also lead to disturbed inflammatory process, enhancing tissue damage in periodontal healing procedure.

Wound healing is a process consisting of overlapping stages, including clot formation, inflammation, proliferation, and remodeling [41, 42]. bFGF has been proven that it can significantly promote the last three stages of wound healing process. Normally, bFGF is produced primarily by periodontal ligament fibroblasts and endothelial cells in the periodontal tissue. In the healing stage of periodontitis, the basic cellular mechanisms of periodontal regeneration are similar to wound healing and involve proliferation and migration of fibroblasts in the periodontal ligament, the differentiation of osteoblasts and cementoblasts from undifferentiated precursor cells, and ultimately, the synthesis of extracellular matrix [43, 44]. bFGF has been shown to be effective in promoting periodontal regeneration in a series of studies [10, 11, 16, 17], whereas the role bFGF plays in the development and recovery stage of periodontitis healing still keeps unknown. It was reported that bFGF levels may be decreased in tissue associated with chronic periodontal lesions, which could be related with reduced secretion of bFGF caused by periodontal ligament cells and endothelial cell damage during periodontitis [34]. Similarly, we also found the decreased bFGF secretion in periodontitis tissue, accompanied by an upside-down ratio between the inflammatory cytokines and bFGF secretion. We considered that it was associated not only with periodontal cell damage during periodontitis but also with the pathological feature of periodontitis, apparent failure of recruitment and activation of macrophage [35]. During healing process, bFGF returned to control level, with which periodontal tissue repair and regeneration gradually took place and the inflammatory factors gradually fell back to slightly higher level compared to the control. It meant the reestablishment of inflammation and repair process homeostasis in the new level. Negative correlation between bFGF and inflammatory factors during periodontitis confirmed by the present study further demonstrated the important roles that bFGF may play in the periodontal healing process. 

Previous studies have shown the negative effects of psychological stress on wound healing. Bosch et al. [21] confirmed a slower rate of mucosa healing of the oral hard palate in individuals with dysphoria compared with the median healing rate, suggesting delayed wound healing under stressed conditions. In addition, it was reported that delayed migration and differentiation of fibroblasts to smooth muscle myofibroblasts may contribute to the prolonged healing time of wounds on the backs of restraint-stressed mice [45]. In the present study, the stressed rats showed significantly impaired periodontal healing and regeneration, along with a significant reduction in the number of bFGF-immunoreactive cells and bFGF staining intensity in the periodontal ligament compared with rats in the natural periodontitis healing group, indicating that psychological stress may delay periodontal healing through downregulation of bFGF expression, which is an essential factor in periodontal regeneration. 

## 5. Conclusions

Psychological stress leads to a delay of periodontal healing in conditions of experimental periodontitis, which may be mediated in part by a downexpression of bFGF in periodontal ligament.

## References

[1] Saini R, Marawar P, Shete S, Saini S, Mani A (2009). Dental expression and role in palliative treatment. *Indian Journal of Palliative Care*.

[2] Van Dyke TE, Dave S (2005). Risk factors for periodontitis. *Journal of the International Academy of Periodontology*.

[3] Hugoson A, Ljungquist B, Breivik T (2002). The relationship of some negative events and psychological factors to periodontal disease in an adult Swedish population 50 to 80 years of age. *Journal of Clinical Periodontology*.

[4] Pistorius A, Krahwinkel T, Willershausen B, Boekstegen C (2002). Relationship between stress factors and periodontal disease. *European Journal of Medical Research*.

[5] Wimmer G, Janda M, Wieselmann-Penkner K, Jakse N, Polansky R, Pertl C (2002). Coping with stress: its influence on periodontal disease. *Journal of Periodontology*.

[6] Van Dyke TE, Serhan CN (2003). Resolution of inflammation: a new paradigm for the pathogenesis of periodontal diseases. *Journal of Dental Research*.

[7] Camargo PM, Lekovic V, Weinlaender M, Vasilic N, Madzarevic M, Kenney EB (2002). Platelet-rich plasma and bovine porous bone mineral combined with guided tissue regeneration in the treatment of intrabony defects in humans. *Journal of Periodontal Research*.

[8] Fentoğlu O, Kirzioğlu FY, Ozdem M, Koçak H, Sütçü R, Sert T (2012). Proinflammatory cytokine levels in hyperlipidemic patients with periodontitis after periodontal treatment. *Oral Diseases*.

[9] Varga J, Domokos A, Barna I, Jankord R, Bagdy G, Zelena D (2011). Lack of vasopressin does not prevent the behavioural and endocrine changes induced by chronic unpredictable stress. *Brain Research Bulletin*.

[10] Takayama S, Murakami S, Mlki Y (1997). Effects of basic fibroblast growth factor on human periodontal ligament cells. *Journal of Periodontal Research*.

[11] Murakami S, Takayama S, Ikezawa K (1999). Regeneration of periodontal tissues by basic fibroblast growth factor. *Journal of Periodontal Research*.

[12] Lalani Z, Wong M, Brey EM (2005). Spatial and temporal localization of FGF-2 and VEGF in healing tooth extraction sockets in a rabbit model. *Journal of Oral and Maxillofacial Surgery*.

[13] Katayama A, Ota M, Sugito H, Shibukawa Y, Yamada S (2006). Effect of proliferating tissue on transplanted teeth in dogs. *Oral Surgery, Oral Medicine, Oral Pathology, Oral Radiology and Endodontology*.

[14] Gospodarowicz D (1974). Localisation of a fibroblast growth factor and its effect along and with hydrocortisone on 3T3 cell growth. *Nature*.

[15] Shimabukuro Y, Ichikawa T, Takayama S (2005). Fibroblast growth factor-2 regulates the synthesis of hyaluronan by human periodontal ligament cells. *Journal of Cellular Physiology*.

[16] Takayama S, Murakami S, Shimabukuro Y, Kitamura M, Okada H (2001). Periodontal regeneration by FGF-2 (bFGF) in primate models. *Journal of Dental Research*.

[17] Murakami S, Takayama S, Kitamura M (2003). Recombinant human basic fibroblast growth factor (bFGF) stimulates periodontal regeneration in class II furcation defects created in beagle dogs. *Journal of Periodontal Research*.

[18] Breivik T, Opstad PK, Gjermo P, Thrane PS (2000). Effects of hypothalamic-pituitary-adrenal axis reactivity on periodontal tissue destruction in rats. *European Journal of Oral Sciences*.

[19] Gaspersic R, Stiblar-Martincic D, Skaleric U (2002). Influence of restraint stress on ligature-induced periodontitis in rats. *European Journal of Oral Sciences*.

[20] Breivik T, Gundersen Y, Osmundsen H, Fonnum F, Opstad PK (2006). Neonatal dexamethasone and chronic tianeptine treatment inhibit ligature-induced periodontitis in adult rats. *Journal of Periodontal Research*.

[21] Bosch JA, Engeland CG, Cacioppo JT, Marucha PT (2007). Depressive symptoms predict mucosal wound healing. *Psychosomatic Medicine*.

[22] Horan MP, Quan N, Subramanian SV, Strauch AR, Gajendrareddy PK, Marucha PT (2005). Impaired wound contraction and delayed myofibroblast differentiation in restraint-stressed mice. *Brain, Behavior, and Immunity*.

[23] Luo KR, Hong CJ, Liou YJ, Hou SJ, Huang YH, Tsai SJ (2010). Differential regulation of neurotrophin S100B and BDNF in two rat models of depression. *Progress in Neuro-Psychopharmacology and Biological Psychiatry*.

[24] Yang C, Wang G, Wang H, Liu Z, Wang X (2009). Cytoskeletal alterations in rat hippocampus following chronic unpredictable mild stress and re-exposure to acute and chronic unpredictable mild stress. *Behavioural Brain Research*.

[25] Xu D, Buehner A, Xu J (2006). A polymorphic glucocorticoid receptor in a mouse population may explain inherited altered stress response and increased anxiety-type behaviors. *The FASEB Journal*.

[26] Zhang Y, Gu F, Chen J, Dong W (2010). Chronic antidepressant administration alleviates frontal and hippocampal BDNF deficits in CUMS rat. *Brain Research*.

[27] Sakoa E, Hosomichib J (2010). Alteration of bFGF expression with growth and age in rat molar periodontal ligament. *The Angle Orthodontist*.

[28] Hu FW, Hosomichi J, Kanno Z, Soma K (2008). The influence of occlusal stimuli on basic fibroblast growth factor expression in the periodontal healing of replanted teeth. *Journal of Medical and Dental Sciences*.

[29] Wang XJ, Liu YF, Wang QY (2010). Functional expression of *α*7 nicotinic acetylcholine receptors in human periodontal ligament fibroblasts and rat periodontal tissues. *Cell and Tissue Research*.

[30] Peruzzo DC, Benatti BB, Antunes IB (2008). Chronic stress may modulate periodontal disease: a study in rats. *Journal of Periodontology*.

[31] Di Paola R, Briguglio F, Paterniti I (2011). Emerging role of PPAR-*β*/*δ* in inflammatory process associated to experimental periodontitis. *Mediators of Inflammation*.

[32] Graves DT, Fine D, Teng YTA, Van Dyke TE, Hajishengallis G (2008). The use of rodent models to investigate host-bacteria interactions related to periodontal diseases. *Journal of Clinical Periodontology*.

[33] Huang S, Lu F, Zhang Z, Yang X, Chen Y (2011). The role of psychologic stress-induced hypoxia-inducible factor-1*α* in rat experimental periodontitis. *Journal of Periodontology*.

[34] Gao J, Jordan TW, Cutress TW (1996). Immunolocalization of basic fibroblast growth factor (bFGF) in human periodontal ligament (PDL) tissue. *Journal of Periodontal Research*.

[35] Chapple CC, Srivastava M, Hunter N (1998). Failure of macrophage activation in destructive periodontal disease. *Journal of Pathology*.

[36] Yuan K, Jin YT, Lin MT (2000). The detection and comparison of angiogenesis-associated factors in pyogenic granulome by immunohistochemistry. *Journal of Periodontology*.

[37] Yang C, Wang G, Wang H, Liu Z, Wang X (2009). Cytoskeletal alterations in rat hippocampus following chronic unpredictable mild stress and re-exposure to acute and chronic unpredictable mild stress. *Behavioural Brain Research*.

[38] Coimbra LS, Rossa C, Guimarães MR (2011). Influence of antiplatelet drugs in the pathogenesis of experimental periodontitis and periodontal repair in rats. *Journal of Periodontology*.

[39] Brechter AB, Persson E, Lundgren I, Lerner UH (2008). Kinin B1 and B2 receptor expression in osteoblasts and fibroblasts is enhanced by interleukin-1 and tumour necrosis factor-*α*. Effects dependent on activation of NF-*κ*B and MAP kinases. *Bone*.

[40] Lv X, Li Q, Wu S, Sun J, Zhang M, Chen YJ (2012). Psychological stress alters the ultrastructure and increases IL-1*β* and TNF-*α* in mandibular condylar cartilage. *Brazilian Journal of Medical and Biological Research*.

[41] Thompson SK, Chang EY, Jobe BA (2006). Clinical review: healing in gastrointestinal anastomoses, part I. *Microsurgery*.

[42] Park NY, Valacchi G, Lim Y (2010). Effect of dietary conjugated linoleic acid supplementation on early inflammatory responses during cutaneous wound healing. *Mediators of Inflammation*.

[43] Bartold PM, Harkin DG, Bignold LP (1989). Proteoglycans synthesized by human polymorphonuclear leucocytes in vitro. *Immunology and Cell Biology*.

[44] Christgau M, Moder D, Hiller KA, Dada A, Schmitz G, Schmalz G (2006). Growth factors and cytokines in autologous platelet concentrate and their correlation to periodontal regeneration outcomes. *Journal of Clinical Periodontology*.

[45] Horan MP, Quan N, Subramanian SV, Strauch AR, Gajendrareddy PK, Marucha PT (2005). Impaired wound contraction and delayed myofibroblast differentiation in restraint-stressed mice. *Brain, Behavior, and Immunity*.

